# Supplementary material for: Behind the Scenes of COVID-19 Vaccine Hesitancy: Psychological Predictors in an Italian Community Sample
Source: Vaccines (Basel). 2022 Jul 21;10(7):1158. doi: 10.3390/vaccines10071158 (PMC9325138; doi:10.3390/vaccines10071158)
Supplement: Supplementary file 1 [file vaccines-10-01158-s001.zip › vaccines-1806165-supplementary.pdf]

Behind the Scenes of COVID-19 Vaccine Hesitancy: Psychological Predictors in an Italian Community Sample

Sofia Tagini, Agostino Brugnera, Roberta Ferrucci, Alberto Priori, Angelo Compare, Laura Parolin, Gabriella Pravettoni, Vincenzo Silani, and Barbara Poletti

Table S1. English translation of the web survey on COVID-19 vaccine acceptance, conducted between April and May 2021 in Italy. For details on the standardized instruments included see the referenced validations.

Section 1. Sociodemographic information

1. Age (open)
2. Sex (select)
   Female
   Male
   I prefer not to report
3. Place of living (open)
4. Marital Status (select)
   Single
   In a relationship
5. Number of Children (open)
6. Education (select)
   Elementary school
   Secondary school
   High School
   Bachelor's degree
   Master's degree
   PhD or other post-lauream specializations
7. Profession (open)
8. If you are a healthcare worker, do/did you work in a COVID-19 ward? (select)
   Yes
   No
9. Annual income (select)
   Low
   Medium
   High

10. With which political wing do you identify most? (select)
    Left
    Center-left
    Center
    Center-right
    Right
    Other (specify)
11. Which is your position concerning Religion? (select)
    Believer (specify)
    Not a believer
    I don't know

12. Section 2. Health status

13. Overall, how would you rate your physical health? (select)
    Excellent
    Good
    Good enough
    Not very good
    Bad
14. Do you suffer from any chronic disease (e.g., diabetes, hypertension, autoimmune diseases) or other relevant clinical conditions? (select)
    Yes (specify)
    No
15. Did you get COVID-19 (i.e., positive swab)? (select)
    Yes, with symptoms
    Yes, with no symptoms
    No

Section 3. Vaccine-related information

16. Do you usually get the flu shot?
    Yes, always
    Sometimes
    No, never
17. Have you ever received other optional vaccinations in adulthood (e.g., HPV, meningococcal, pneumococcal, hepatitis, pertussis)?
    Yes
    No
18. Did you receive COVID-19 vaccine?
    Yes
    No, but I certainly will
    No, I haven't decided yet
    No, and I will certainly not

19. Have you decided to get the shot, or will you do so in the future only because/if forced by law?
    Yes, I did so. or I will only because/if forced
    No, I did so. or I will of my choice
20. Are people important to you (relatives and/or friends) favorable to receive COVID-19 vaccine?
    Yes, very favorable
    Enough favorable
    Not at all favorable
21. How much do you feel informed about COVID-19 vaccine (e.g., concerning its functioning and/or possible side effects)?
    Very Informed
    Informed enough
    Little informed
    Not at all informed
22. Do you think the information received on COVID-19 vaccine is sufficient?
    Yes, totally
    Enough
    Not at all

Section 4. Psychological variables

Risk Perception Questionnaire (answers were given on a 5-point Likert-type scale from 1 = totally disagree to 5 = totally agree).

Severity Item 1. Being infected by COVID-19 can be a very serious matter

Severity Item 2. It can be hard to cure people affected by COVID-19

Severity Item 3. Being infected by COVID-19 can be very dangerous

Vulnerability Item 1. I am likely to be infected by COVID-19

Vulnerability Item 2. It is more likely that I could be infected by COVID-19 than other people of the same age and gender

Psychological Standardized Measures:

5C Psychological Antecedents of Vaccination Scale (5C-PAV; Betsch et al., 2018)

Ten Items Personality Inventory (TIPI; Chiorri et al., 2015)

Health Locus of Control Scale (H-LoC; Donizzetti & Petrillo, 2015)

Conspiracy Mentality Questionnaire (CMQ; Bruder et al., 2013)

The Portrait Values Questionnaire (PVQ-21; Schwartz, 2003)

Table S2. Italian translation of the 5C Psychological Antecedents of Vaccination scale (5C-PAV; Betsch et al., 2018).

Per favore, indichi quanto è d'accordo con le seguenti affermazioni

Confidence

1) Sono assolutamente fiducioso che il vaccino contro il COVID-19 sia sicuro
2) La vaccinazione control il COVID-19 è efficace
3) Per quanto riguarda il vaccino control il COVID-19, sono sicuro che le autorità pubbliche decidano nel massimo interesse della comunità

Complacency

4) La vaccinazione control il COVID-19 non è necessaria, poiché non è comune contrarre la malattia
5) Il mio sistema immunitario è così forte che mi protegge dal COVID-19
6) Il COVID-19 non è tanto grave che dovrei vaccinarmi

Constraints

7) Le incombenze di vita quotidiana mi impediscono di vaccinarmi control il COVID-19
8) Per me è scomodo fare la vaccinazione per COVID-19
9) Andare dal dottore mi mette a disagio; questo mi trattiene dal vaccinarmi control il COVID-19

Calculation

10) Nel valutare di essere vaccinato control il COVID-19, faccio un bilancio dei benefici e dei rischi per prendere la migliore decisione possibile
11) Per decidere se vaccinarmi contro il COVID-19, considero attentamente se mi è utile
12) Per me è importante comprendere appieno ciò che riguarda la vaccinazione contro il COVID-19 prima di vaccinarmi

Collective responsibility

13) Se tutti sono vaccinati contro il COVID-19, non devo vaccinarmi anche io (R)
14) Mi vaccino contro COVID-19 perché così posso proteggere anche le persone con un sistema immunitario più debole
15) La vaccinazione contro COVID-19 è un'azione collettiva per ostacolare la diffusione di una malattia

Opzioni di Risposta:

Fortemente in disaccordo, moderatamente in disaccordo, leggermente in disaccordo, né in accordo né in disaccordo, leggermente d'accordo, moderatamente d'accordo, fortemente d'accordo

Note. (R) indicate reverse items.

Table S3. Italian translation of the Conspiracy Mentality Questionnaire (CMQ; Bruder et al., 2013).

Legga attentamente ciascuna affermazione e selezioni la percentuale che ritiene rappresenti meglio il grado di probabilità che ritiene valido

Penso che…

1) ... nel mondo accadano molte cose molto importanti, di cui il popolo non viene mai informato.
2) … i politici di solito non ci dicano i veri motivi delle loro decisioni.
3) … le agenzie governative monitorino da vicino tutti i cittadini.
4) … eventi che superficialmente sembrano mancare di connessione sono spesso il risultato di attività segrete.
5) … ci sono organizzazioni segrete che influenzano notevolmente le decisioni politiche.

Opzioni di Risposta:

Certamente no 0% • Estremamente Improbabile 10% • Molto Improbabile 20% • Improbabile 30% • Alquanto Improbabile 40% • Indeciso 50% •Alquanto Probabile 60% • Probabile 70% • Molto Probabile 80% •Estremamente Probabile 90% •Certamente Sì 100%

Results of the Confirmatory Factorial Analysis (CFA) on the Italian versions of 5C-PAV and CMQ scales

To examine the internal validity of both the 5C-PAV and CMQ scales, we ran two separate Confirmatory Factorial Analyses (CFA) using Analysis of Moment Structures (AMOS) version 26.0. We first examined the presence of univariate and multivariate outliers and tested if items of both scales were normally distributed (i.e., with a skewness < |2| and a kurtosis < |7|; Byrne, 2010). As for the CFA, parameter estimates were computed using a maximum likelihood estimation method, while an optimal model fit was evaluated using the following criteria: a root mean square error of approximation (RMSEA) of 0.05 or less, an upper RMSEA's 90% confidence interval bound of 0.08 or less, a comparative fit index (CFI) and a Tucker-Lewis index (TLI) of 0.95 or more, and a standardized root mean squared residual (SRMR) of 0.05 or less. The magnitude of factor loadings was interpreted according to Cohen's criteria.

As for the 5C-PAV, several items (4, 6, 7, 9, 13, 14 and 15) were positively or negatively skewed severely and were transformed with an inverse or a reflect and inverse transformation (Tabachnick & Fidell, 2007). No univariate outliers were identified, however we found 60 multivariate outliers which were removed from subsequent analyses. The CFA evidenced a good fit to the data, χ^2 (80) = 184.509, $p < 0.001$; RMSEA = 0.036 (90% CI: 0.029 - 0.034); CFI = 0.98; TLI = 0.98; SRMR = 0.036. Finally, factor loadings ranged from .48 to .92, with medium to large effects (see Supplementary Table S4 for details).

As for the CMQ, all items were normally distributed. No univariate outliers were identified, however we found 18 multivariate outliers which were removed from subsequent analyses. After modeling a covariance between the error terms of Item 1 and 2 (r = .46, $p < .001$), the CFA evidenced an adequate fit to the data, χ^2 (4) = 5.985, $p < 0.001$; RMSEA = 0.069 (90% CI: 0.044 - 0.096); CFI = 0.99; TLI = 0.98; SRMR = 0.018. Finally, factor loadings ranged from .55 to .91, with large effects (see Supplementary Table S5 for details).

Table S4. Factor Loadings for the 5C-PAV subscales (n = 1019).

5C-PAV subscale	Item #	Factor Loading
Confidence	Item 1	.874*
\	Item 2	.923*
\	Item 3	.681*
Complacency	Item 4[a]	.837*
\	Item 5	.526*
\	Item 6[a]	.860*
Constraints	Item 7[a]	.659*
\	Item 8	.495*
\	Item 9[a]	.691*
Calculation	Item 10	.721*
\	Item 11	.749*
\	Item 12	.616*
Collective Responsibility	Item 13[a]	.480*
\	Item 14[a]	.788*
\	Item 15[a]	.914*

Note. 5C-PAV = Psychological Antecedents of Vaccination scale. [a] = Item transformed with an inverse or a reflect and inverse transformation (the direction of the effect was changed to positive for ease of interpretation). * $p < .001$

Table S5. Factor Loadings for the Conspiracy Mentality Questionnaire (CMQ; $n = 1061$).

**Item #**	**Factor Loading**
Item 1	$.550^{*}$
Item 2	$.601^{*}$
Item 3	$.691^{*}$
Item 4	$.907^{*}$
Item 5	$.875^{*}$

Note. $^{*}\ p < .001$
